# The Evolution of Immunosuppressive Therapy in Pig-to-Nonhuman Primate Organ Transplantation

**DOI:** 10.3389/ti.2024.13942

**Published:** 2025-01-13

**Authors:** S. A. Sanatkar, K. Kinoshita, A. Maenaka, H. Hara, D. K. C. Cooper

**Affiliations:** ^1^ Center for Transplantation Sciences, Department of Surgery, Massachusetts General Hospital, Harvard Medical School, Boston, MA, United States; ^2^ The Transplantation Institute at the Second Affiliated Hospital of Hainan Medical University, Haikou, Hainan, China

**Keywords:** immunosuppression, xenotransplantation, transplantation immunology, swine, non-human primate

## Abstract

An overview is provided of the evolution of strategies towards xenotransplantation during the past almost 40 years, focusing on advances in gene-editing of the organ-source pigs, pre-transplant treatment of the recipient, immunosuppressive protocols, and adjunctive therapy. Despite initial challenges, including hyperacute rejection resulting from natural (preformed) antibody binding and complement activation, significant progress has been made through gene editing of the organ-source pigs and refinement of immunosuppressive regimens. Major steps were the identification and deletion of expression of the three known glycan xenoantigens on pig vascular endothelial cells, the transgenic expression of human “protective” proteins, e.g., complement-regulatory, coagulation-regulatory, and anti-inflammatory proteins, and the administration of an immunosuppressive regimen based on blockade of the CD40/CD154 T cell co-stimulation pathway. Efforts to address systemic inflammation followed. The synergy between gene editing and judicious immunomodulation appears to largely prevent graft rejection and is associated with a relatively good safety profile. Though there remains an incidence of severe or persistent proteinuria (nephrotic syndrome) in a minority of cases. This progress offers renewed hope for patients in need of life-saving organ transplants.

## Introduction

Despite the great progress that has been made in the gene-editing of pigs that are the sources of organs or cells for xenotransplantation in nonhuman primate (NHP) or human recipients, there remains a need for the administration of exogenous immunosuppressive therapy to the recipient of a pig xenograft [1]. Increased gene-editing of the organ-source pigs [2, 3] and the introduction of new agents that are more effective in suppressing the human immune response are both key factors that have allowed changes to be made to the immunosuppressive regimen to prevent rejection.

We here briefly review the changes in pre-transplant treatment, immunosuppressive protocols, and adjunctive therapy that have been made during the past almost 40 years of pig-to-NHP heart or kidney transplantation with some based on concomitant *in vitro* studies [4]. These observations are made largely through the experience of one group but that of several other groups has also been reviewed.

## The “Conventional” Immunosuppressive Therapy Era

By the 1980s, it was known that natural (preformed) antibodies, when bound to antigens on a discordant animal organ graft, could activate complement, resulting in hyperacute rejection (defined as rejection occurring within 24 h) [5–10]. There was evidence that natural antibodies developed as a defense mechanism when the gastrointestinal tract of infants became colonized by microorganisms that expressed carbohydrate antigens, e.g., galactose-α1,3-galactose (Gal), that were also expressed on pig cells (Table 1) [11, 12].

**TABLE 1:** Glycan xenoantigens that have been deleted in gene-edited pigs.

Carbohydrate (abbreviation)	Responsible enzyme	Gene-knockout pig
1. Galactose-α1,3-galactose (Gal)	α1,3-galactosyltransferase	GTKO
2. N-glycolylneuraminic acid (Neu5Gc)	Cytidine monophosphate-N-acetylneuraminic acid hydroxylase (CMAH)	CMAH-KO
3. Sda	β-1,4N-acetylgalactosaminyltransferase	β4GalNT2-KO

### Antibody Depletion

When xenotransplantation was first explored in wild-type (i.e., genetically-*unmodified*) pig-to-NHP models in the 1980s [13, 14], cyclosporine had become available, but tacrolimus was not yet accessible to most groups. Initial studies were therefore based on the regimens used in clinical allotransplantation, i.e., cyclosporine with added steroids with or without azathioprine or mycophenolate mofetil (MMF) (Table 2) [14]. The results were extremely disappointing and, with graft survival still measured in minutes, hours, or a few days, the administration of a cyclosporine-based regimen made little difference to the outcome. The innate immune response was clearly very strong and the effect of cyclosporine in suppressing the adaptive immune response was very modest (in contrast to its effect in allotransplantation).

**TABLE 2:** Representative immunosuppressive regimen administered in the wild-type pig-to-NHP heterotopic heart Tx model [14].

Pre-transplant therapy
*Ex vivo* hemoperfusion of recipient’s blood through a donor-specific pig kidney for 1 h (x2 kidneys)
Splenectomy (in some cases) 4–8 days before the transplant
Induction therapy
Cyclosporine by continuous IV infusion (15–32 mg/kg/day) until a therapeutic level of >400 ng/mL was achieved
Maintenance therapy
Cyclosporine by continuous IV infusion (15–32 mg/kg/day) to maintain therapeutic level of >400 ng/mL
Methylprednisolone 10 mg/kg/day IM (on day of transplant), with plan to taper the dose to 2 mg/kg/day
Outcome
Longest heart graft survival = 5 days

As pre-transplant splenectomy was thought to be beneficial in allotransplantation across the ABO blood group barrier [15, 16], it was believed that it might also reduce the immune response to a pig graft, probably by removing a major source of B cells in the host as well as by decreasing the number of lymphocytes and their proliferative responses [17, 18]. With time, however, evidence for this was lacking and, possibly after the addition of rituximab to the protocol, it was eventually omitted from the regimen.

An effort was made to deplete the potential recipient of anti-pig antibodies either by 1) plasmapheresis [17], which extended graft survival to a maximum of 23 days, though usually for a shorter period of time, or by 2) preliminary perfusion of the recipient blood through a donor-specific second organ, e.g., the kidney, before donor-specific heart transplantation [13, 14], but graft survival remained very limited.

When Gal was clearly identified as the major target for human preformed anti-pig antibodies (Table 1) [19–23], techniques of antibody depletion were explored *in vitro* [24, 25] and refined to allow removal or “neutralization” of only anti-Gal antibodies, thus not depleting the NHP of antibodies that might be important in protecting from infectious complications. Again based on experience in overcoming the barrier of ABO-incompatibility, anti-pig antibody immunoadsorption was achieved by 1) perfusion of the recipient’s blood through an immunoaffinity column of synthetic Gal oligosaccharides [20, 26–33] or 2) the continuous intravenous infusion of soluble synthetic Gal oligosaccharides [33–36].

In this latter approach, the infused synthetic Gal oligosaccharides would be bound by the circulating anti-Gal antibodies and therefore “neutralize” them by preventing them from binding to the pig graft. This approach had proven to be successful in preventing rejection of ABO-incompatible cardiac *allografts* in baboons [14]. Subsequently, modifications were made, e.g., synthetic Gal oligosaccharides were attached to bovine serum albumin which was then infused intravenously [37, 38]. All of these approaches prevented hyperacute rejection of the graft, but the return of antibody within days inevitably resulted in graft loss [39, 40].

In retrospect, the removal or neutralization of anti-Gal antibodies alone was probably misguided because, in addition to the early return of anti-Gal antibodies, there was already evidence of the presence of antibodies to non-Gal antigens [41–43] (identified as N-glycolylneuraminic acid [44] and Sda [45] [Table 1]). However, at the time, it was hoped that “accommodation” would develop [46] (i.e., when the return of antibody is no longer associated with rejection) as occurs in many patients receiving an ABO-incompatible allograft [47], but this proved not to be the case. The exact mechanism by which accommodation occurs remains uncertain. The additional differences in complement and coagulation factors between pig and human (see below) probably contributed to the difference in outcome between allograft and xenograft.

### Protection From Complement Injury

Complement depletion or inhibition in the potential NHP recipient extended graft survival to a maximum of 25 days [48–52], but it was suspected that patients with no complement activity would be at risk for infectious complications and would not do well long-term and so this approach was not pursued (although prolonged complement inhibition has been adopted again by some groups recently [53]).

The introduction of the first gene-edited pigs by White and his colleagues at the British biotechnology company, Imutran, was a milestone in xenotransplantation research and enabled progress to be made [54]. These pigs expressed a single human complement-regulatory protein, CD55 (decay accelerating factor, DAF), and this alone extended kidney or heart graft survival in some immunosuppressed recipient NHPs for several weeks – in one case for up to 3 months [55]. However, the transplantation of hCD55 transgenic pig grafts proved successful only if intensive cyclosporine-based immunosuppressive regimens were employed (Table 3) [56]. For example, induction therapy with cyclophosphamide was found to be beneficial or even essential. It was later confirmed that the expression of a human complement-regulatory protein helps protect the graft from systemic complement activation in the host [57].

**TABLE 3:** Representative immunosuppressive regimen administered in the hDAF (CD55) transgenic pig-to-NHP heterotopic heart and kidney Tx models [55].

Pre-transplant therapy
None
Induction
Cyclophosphamide 40 mg/kg on day −1 IV and 10 mg/kg on day 0 (the day of the transplantation) IV
Cyclosporine 35 mg/kg x2 daily orally from day −2
Methylprednisolone 1 mg/kg IV on day 0
Maintenance
Cyclosporine 35 mg/kg x2 daily orally to achieve a 12-hour trough level of 300–500 ng/mL
Prednisone 1 mg/kg orally on days 1 and 2 with subsequent tapering by 0.05 mg/kg/day to a baseline dose of 0.2 mg/kg/day from day 18
Outcome
Longest life-supporting kidney graft survival = 90 days
Longest non-life-supporting (heterotopic) heart graft survival **=** 62 days

Depletion of complement for a prolonged period of time was, and still is, considered to put the patient at increased risk of infectious complications. Once the transgenic expression of human complement-regulatory proteins could be induced in the organ-source pig, systemic complement inhibition was avoided by most groups. However, transient systemic complement inhibition at the time of pig organ transplantation, when there is complement activation and inflammation, may be beneficial [58]. When introduced by Langin et al [59], the administration of a C1-esterase inhibitor on just 2 days appears to be safe and beneficial, even though it has not been conclusively demonstrated to be essential. Long-term complement inhibition, e.g., with a C5 inhibitor, has been incorporated into the immunosuppressive regimen by some groups [53], but its necessity remains controversial.

## The Introduction of CD40/CD154 T Cell Co-Stimulation Pathway Blockade

Based on encouraging studies in models of allotransplantation, in 2,000 Buhler et al. carried out pig hematopoietic cell transplantation in an attempt to induce chimerism as a basis for achieving immunological tolerance to a pig organ in an immunosuppressed NHP [60, 61]. When immunosuppressive therapy was based on cyclosporine, an elicited antibody response to the pig cells was clearly detected within the first 14 post-transplant days. In contrast, treatment with an anti-CD154 monoclonal antibody (mAb) prevented this antibody response [60] (Blockade of the B7/CD28 pathway did not prove equally successful) [62–65]. This proved a major step forward. Since then, almost all groups have employed an anti-CD154 or anti-CD40mAb as the basis of their immunosuppressive regimen [53, 66–77].

**FIGURE 1:**
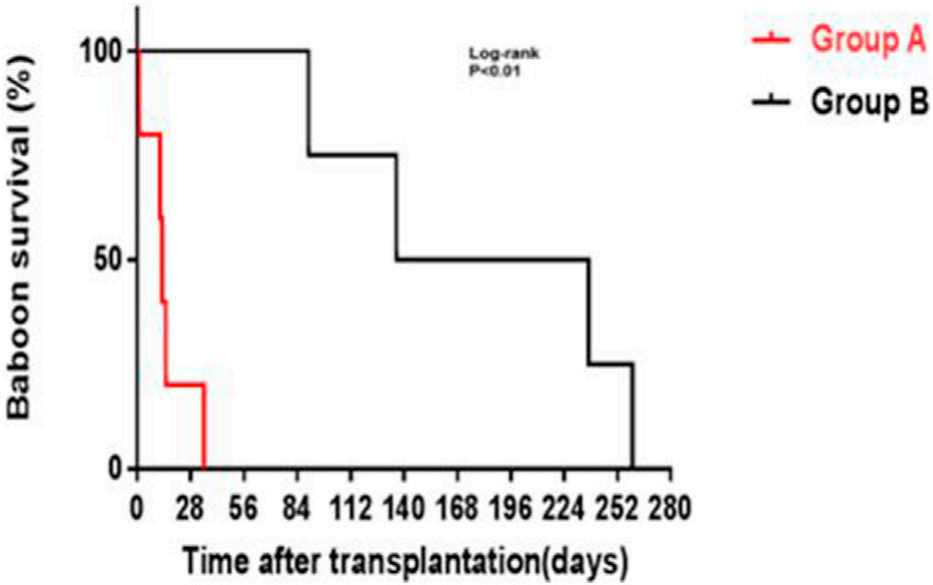
GTKO pig kidney survival in baboons receiving US FDA-approved immunosuppressive agents (Group A, in red) was much shorter than in those receiving an anti-CD40mAb-based regimen (Group B, in black) outlined in Table 6 (Reproduced with permission from Yamamoto T, et al. [81]).

Yamamoto et al. subsequently demonstrated prolonged survival of kidney grafts from α1,3-galactosyltransferase gene-knockout (GTKO) pigs (in which the most important xenoantigen, galactose-α1,3-galactose [Gal] against which humans and NHPs have natural antibodies had been deleted [78–80] in NHPs receiving a CD40/CD154 co-stimulation-based inhibitory regimen compared to NHPs receiving a conventional (tacrolimus-based) immunosuppressive regimen (Figure 1) [81]. The administration of an anti-CD154mAb has been associated with better results when compared with an anti-CD40mAb [74]. In summary, by modulating the immune response between T cells and antigen-presenting cells, inhibiting co-stimulatory pathways improves long-term post-transplant outcomes.

When attempting to induce tolerance to an allotransplant, at that time the potential recipient was pre-treated with whole body and thymic irradiation. However, in the pig-to-NHP model, survival of the graft rather than tolerance induction was the major aim, and so whole body irradiation was deemed unnecessary, although thymic irradiation was still carried out for a period of time (Table 4).

**TABLE 4:** Representative immunosuppressive regimen administered in the GTKO pig-to-NHP heterotopic heart Tx model [82].

Pre-transplant therapy
Thymic irradiation 700 cGy (day −1)
Induction
Anti-thymocyte globulin (horse ATG [ATGAM])* 50 mg/kg IV on days −3, −2, and −1 (3 doses)
LoCD2b** 1–4 mg/kg IV on days 1–7
Cobra venom factor (CVF) 6 mg/day IV for 4–15 days in some cases
Maintenance
Anti-CD154mAb (AB1793, Novartis) 25 mg/kg IV
Mycophenolate mofetil (MMF) 25–110 mg/kg/day by continuous IV infusion from day −2
Methylprednisolone 4 mg/kg IM daily reducing to 0.5 mg/kg/day
Heparin 5–60 U/kg/h IV from day 0
Aspirin 40 mg on alternate days from day 4 (in some cases)
Outcome
Survival from 2 to 6 months (median 78 days)

*To deplete T cells, the necessary dose of horse ATG [ATGAM] is significantly higher than of rabbit ATG (Tables 5, 6).

**LoCD2b depletes cells expressing CD2 that include T and NK cells [157].

Nevertheless, induction therapy was still considered essential. Initially, this consisted of large doses of anti-thymocyte globulin and other agents that depleted T cells (Table 4). With the transplantation of organs from pigs expressing fewer xenoantigens and a greater number of human protective transgenes it was determined that the dose of ATG could be reduced. The effect of the ATG can be determined simply by following the total lymphocyte count.

**FIGURE 2:**
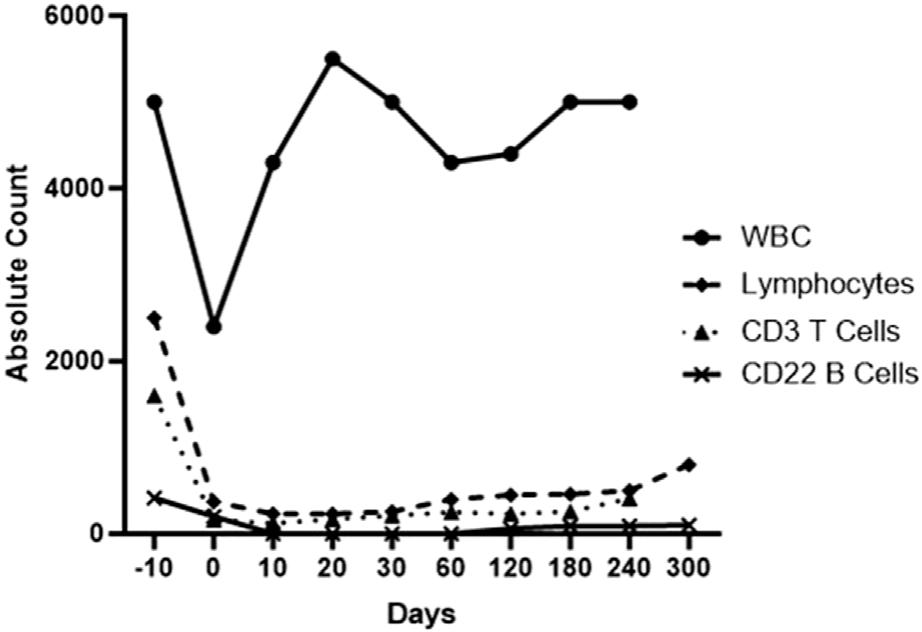
Total white blood cell, lymphocyte, T cell, and B cell counts in the blood of a baboon with a life-supporting pig kidney that received the immunosuppressive regimen outlined in Table 6.

However, additional B cell depletion was considered beneficial and so the administration of an anti-CD20mAb (in the form of Rituximab) was included, initially by McGregor et al [83]. Whether this is essential remains uncertain but a significant reduction in B cells in the blood for several weeks (Figure 2) may possibly result in a subsequent reduction in plasma cells, though this has not been proven.

The initial anti-CD154mAbs were tested *in vitro* and *in vivo* [75, 84–86]. When transplants were still being carried out with organs from wild-type pigs, the results remained disappointing because the innate response remained strong. When GTKO pigs became available, however, the results improved markedly. The first series that combined the transplantation of heterotopically-placed hearts (i.e., *not* life-supporting) from GTKO pigs into baboons with immunosuppression based on CD40/CD154 co-stimulation pathway blockade demonstrated greatly improved graft survival, extending to a maximum of 6 months (Table 4) [82, 87]. However, the recipient baboons were selected on the basis of their low anti-pig antibody levels.

Using an identical immunosuppressive regimen, life-supporting GTKO kidney grafts survived approximately only half as long [88], possibly because 1) kidneys may be more immunogenic than hearts, or 2) the kidneys were life-supporting whereas the hearts were not. When GTKO pig kidneys were transplanted into NHPs immunosuppressed with a tacrolimus-based regimen, the results were markedly inferior [89].

The transplantation of GTKO hearts that expressed a different human complement-regulatory protein, CD46, with the same or very similar immunosuppressive regimen reduced early graft failure but did not extend maximum graft survival [90, 91].

The withdrawal of the original anti-CD154mAbs because of their thrombogenic effect [92–94] necessitated the use of anti-CD40mAbs, first introduced into xenotransplantation by Mohiuddin et al [68–70]. However, increasing data indicate that anti-CD154 agents are superior to anti-CD40 agents in preventing both the adaptive immune response and some aspects of the innate response [75, 95]. Once Fc-modified anti-CD154 agents (that do *not* result in platelet activation) were introduced [74, 75, 96–98], these soon became the treatment of choice [74, 76, 77].

One important observation made in regard to anti-CD154mAb therapy was that in infant baboons in which natural antibodies had not yet developed, treatment with an anti-CD154mAb prevented the development of natural anti-Gal and anti-AB antibodies, suggesting that natural antibodies may be, at least in part, T cell-dependent [99]. This has considerable relevance to the treatment of neonates with complex, life-threatening congenital heart disease, e.g., single ventricle physiology, by pig heart xenotransplantation [100, 101]. For example, by inhibiting both natural and elicited antibody production, treatment with an anti-CD154mAb during the first week of life (at the time of pig heart transplantation) might possibly facilitate the development of immunological tolerance to the graft. Once the graft has been established, it may be possible to discontinue all immunosuppressive therapy.

**FIGURE 3:**
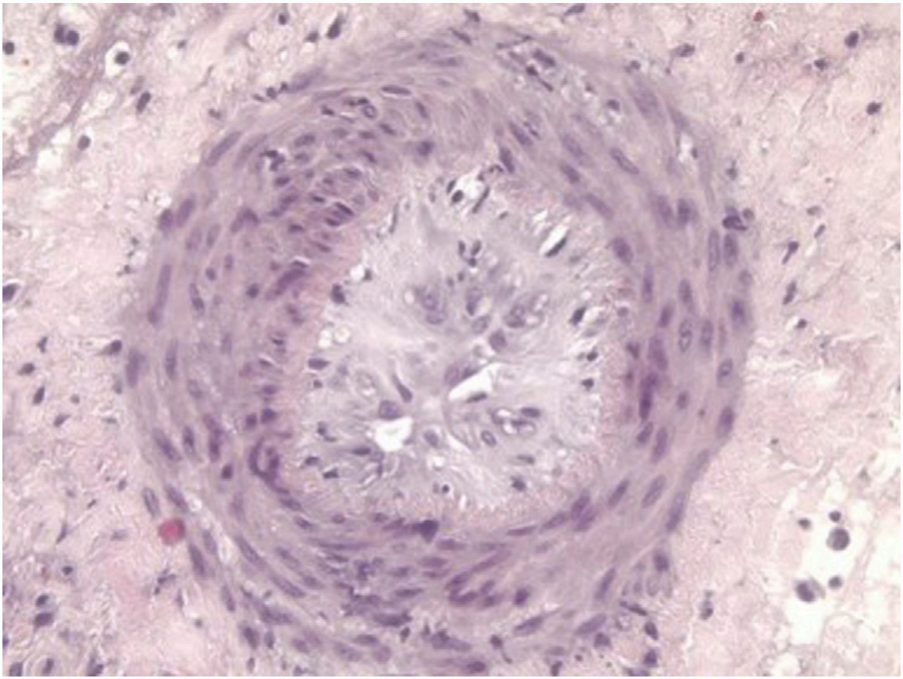
Histopathological features of graft vasculopathy (chronic rejection) In a GTKO pig heart transplanted heterotopically (in the abdomen) 3 months previously in a baboon that received the immunosuppressive regimen outlined in Table 4.

Despite the suppressive effect of agents that block the CD40/CD154 co-stimulation pathway, the transplantation of kidneys or hearts from GTKO pigs was not consistently successful, with some grafts failing from antibody-mediated rejection [66, 82, 88, 102–105], coagulation dysfunction, or graft vasculopathy (chronic rejection) (Figure 3) [106].

### Protection From Coagulation Dysfunction

**FIGURE 4:**
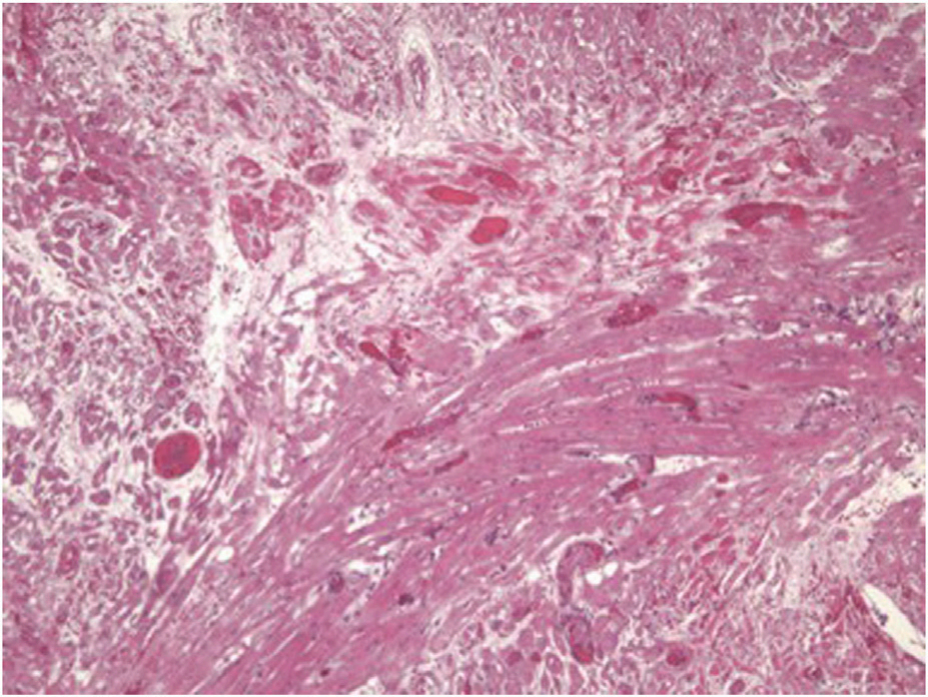
Histopathological features of a pig cardiac graft demonstrating multiple vascular thromboses with surrounding ischemic changes (fibrosis) in a baboon that received the immunosuppressive regimen outlined in Table 4.

Predictions of significant differences in the coagulation-anticoagulation systems between pig and human had been discussed for some time [107, 108], but evidence in the important pig-to-NHP model was first reported in the late 1990s [84, 109–111] (Figure 4). Although the presence of porcine cytomegalovirus (CMV) in the graft was identified as playing a role in coagulation dysfunction [112], later confirmed by Yamada [113], this problem stimulated the need to introduce human coagulation-regulatory genes into the pig. Thrombomodulin, endothelial cell protein C receptor (EPCR), and/or tissue factor pathway inhibitor (TFPI) were expressed in the pig.

**FIGURE 5:**
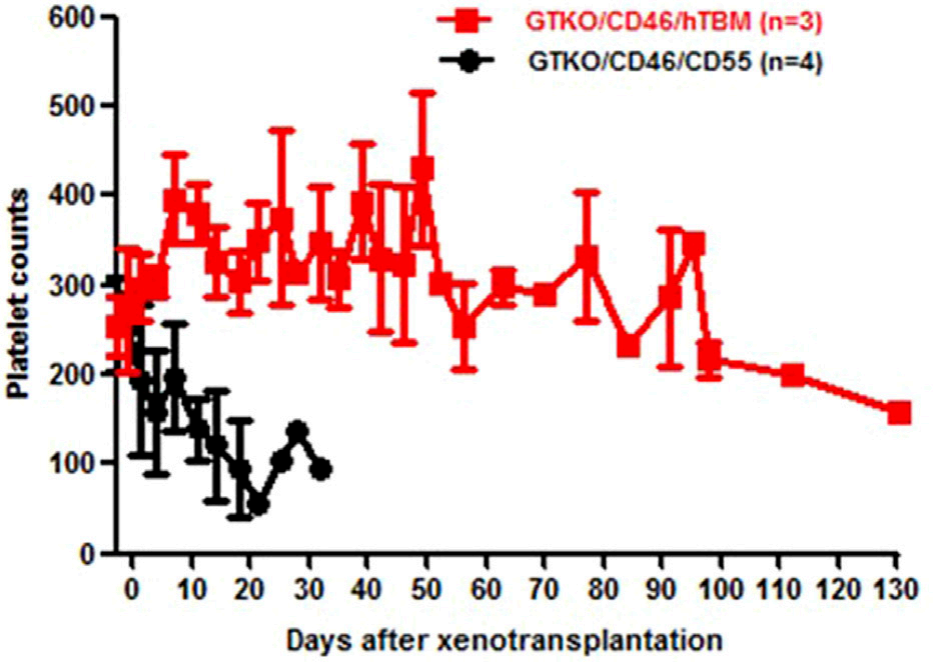
Platelet counts in baboons with hearts from GTKO.CD45.TBM (red) or GTKO.CD46.CD55 (black) pigs. The baboons received the immunosuppressive regimen outlined in Table 5.

When the problems relating to coagulation dysfunction between pig and human were confirmed, the introduction of GTKO. hCD46 pigs that additionally expressed human thrombomodulin reduced the incidence of thrombotic microangiopathy in the pig graft and of consumptive coagulopathy in the recipient NHPs (Table 5; Figure 5) [70]. This prolonged life-supporting kidney graft survival to 7–8 months, with termination of the experiments from infectious complications rather than from rejection [70].

**TABLE 5:** Representative immunosuppressive regimen used in the GTKO/CD46/hTBM pig-to-NHP life-supporting kidney Tx model [70].

Pretransplant therapy
None
Induction
Anti-thymocyte globulin (ATG) 5 mg/kg IV on day −2 (i.e., 2 days before kidney transplantation)
Anti-CD20 mAb (Rituximab) 10 mg/kg IV on day −1
C1 esterase inhibitor 17.5 units/kg IV on days 0 and 2
Maintenance
Anti-CD40 mAb 20 mg/kg IV on days 0, 2, 7, 10, 14, and weekly
Rapamycin x2 daily IM to maintain a 12-hour trough level of 6–12 ng/mL
Methylprednisolone 10 mg/kg IV tapering the dose over the first week to 0.25 mg/kg IM daily
Anti-TNF mAb (etanercept) (in some cases)
Tocilizumab (IL-6R blockade) 8 mg/kg IV monthly for 6 months
Outcome
Two grafts functioned for >7 and >8 months, respectively, with the experiments being terminated for infectious complications
(When human thrombomodulin (hTBM) was *not* expressed in the kidney, a consumptive coagulopathy developed within 12 days, necessitating euthanasia)

### Protection From Systemic Inflammation

Xenotransplantation was found to be accompanied by a systemic inflammatory response that could be detrimental to the survival of the graft by augmenting the immune response and/or coagulation dysfunction [62, 114–118]. Corticosteroids appeared to have no effect in suppressing this response.

However, in the period 2015-2020, the beneficial effects of interleukin-6 receptor (IL-6R) blockade using tocilizumab (which blocks IL-6 binding to the receptors on NHP cells but *not* on pig cells) were investigated [119–121]. Although the initial results were encouraging, the accompanying rise in IL-6 in the blood engendered some caution in the use of tocilizumab [120, 121]. With additional experience, we have tentatively concluded that this agent has a positive effect on graft survival [77]. It may be particularly beneficial when orthotopic pig heart transplantation is carried out because it may protect the *recipient’s t*issues, e.g., the lungs, from inflammatory injury associated in part to the need for cardiopulmonary bypass [118]. However, the combination of two inhibitors of IL-6 proved fatal from profound thrombocytopenia [122].

There is some evidence that the introduction of a human “anti-inflammatory” transgene into the pig, e.g., hemeoxygenase-1, A20, has a protective effect on the graft [3, 121, 123].

Low-dose corticosteroids have been used in almost all regimens (probably because they are included in the regimens of most patients with organ allografts) but there is little evidence that they are essential when co-stimulation blockade is employed, particularly if tocilizumab is being administered. As long ago as 2005, Yamada carried out one GTKO pig kidney transplant in a baboon without maintenance steroids and found no significant detrimental effect on graft survival [88]. Our suspicion is that steroids add little to the efficacy of the regimen, particularly if it includes tocilizumab.

Although not fully recognized in the early days of xenotransplantation research, inhibition of complement activity also has beneficial effects on coagulation dysfunction and the inflammatory response (see below) [58].

## Additional Immunosuppressive Therapy and Adjunctive Therapy

Although blockade of the CD40/CD154 co-stimulation pathway has formed the basis of all effective regimens for the past two decades [60], its dosage is important. For example, dosing of the Tonix-1500 anti-CD154mAb at 20 mg/kg weekly, although effective in preventing rejection of allografts, was not entirely successful in regard to grafts from triple-knockout (TKO) pigs, in which expression of all three of the known pig xenoantigens against which humans have natural antibodies has been deleted (Table 1) [74]. A higher dosage, however, appears to be consistently successful without significant infectious complications [77] and Kinoshital et al. (unpublished data).

It must be remembered that *all* Old World NHPs have natural antibodies to TKO pig cells, thus increasing the hurdle that has to be overcome, i.e., the hurdle of “sensitization” (that will *not* be the case in many human patients receiving a pig xenograft) [124–127]. The results of TKO pig organ transplantation in NHPs are inferior to those of GTKO pig organ transplantation [71, 128], but remarkably CD40/CD154 co-stimulation pathway blockade appears to overcome this hurdle if recipient NHPs are selected with low anti-pig antibody levels (Table 6) [72, 77, 129].

**TABLE 6:** Representative immunosuppressive regimen used in the TKO (+added transgenes) pig-to-NHP life-supporting kidney Tx model [77].

Pretransplant therapy
None
Induction
Anti-thymocyte globulin (ATG), 5 mg/kg IV on day −2 (i.e., 2 days before kidney transplantation [day 0])
Anti-CD20mAb (Rituximab) 10 mg/kg IV on day −1
C1 esterase inhibitor 17.5 units/kg IV on days 0 and 2
Maintenance
Anti-CD154mAb (Tonix-1500), 30 mg/kg IV on days 0, 2, 7, 10, 14, and weekly
Rapamycin daily IM to maintain a 24-hour trough level of 8–12 ng/mL
Methylprednisolone 10 mg/kg IV tapering the dose over the first week to 0.25 mg/kg IM daily
Tocilizumab (IL-6R blockade) 8 mg/kg IV monthly for 6 months
Outcome
Maximum ongoing graft function is now >12 months

Whether other agents, if any, need to be combined with co-stimulation blockade remains uncertain. Pierson and Kawai and their respective colleagues have clearly demonstrated that in *allo*transplantation no other agents (either for induction or maintenance) may be necessary because anti-CD154mAb alone (in the form of Tonix-1500) prevents rejection almost consistently for at least 6 months (at which time the studies have been electively concluded) [97, 98]. Rejection develops only some weeks or months after cessation of treatment. There is some evidence, however, that the addition of low-dose rapamycin or tacrolimus to the regimen ensures an absence of rejection.

However, these agents alone are not so effective in xenotransplantation. For example, Tonix-1500 alone (with *no* induction therapy or additional maintenance therapy) was associated with antibody-mediated rejection of a kidney graft from a pig with 10 gene-edits on post-transplant day 4 [77]. This indicated to us that some form of induction therapy and additional maintenance therapy is required (Table 6).

When both T and B cells are depleted by this induction therapy (Table 6), the anti-CD40 or anti-CD154mAb maintenance therapy appears to maintain low lymphocyte counts throughout the first 6 months of the post-transplant period (Figure 2), which we suspect contributes to preventing an immune response to the graft [70, 77, 130].

To augment the effect of anti-CD40 or anti-CD154mAbs, we have selected rapamycin, in part because it can be administered intramuscularly, which is an advantage when managing NHPs that do not consistently take oral medications [131]. Mammalian target of rapamycin (mTOR) inhibitors have several properties that may be especially beneficial in xenotransplantation, e.g., suppression of T cell proliferation, increases in the number of T regulatory cells, inhibition of pig graft growth, and anti-inflammatory, anti-viral, and anti-cancer effects [132].

However, rapamycin is not tolerated by some patients (largely from gastrointestinal disturbances or oral ulcers) and so other pharmacologic immunosuppressive agents have been incorporated in several regimens. MMF has perhaps been the most commonly used agent [74, 76] but its value, as with rapamycin and tacrolimus, has *not* been proven. No group has yet had the courage to maintain immunosuppression with co-stimulation blockade alone. However, preliminary evidence that this may be possible was reported in one baboon when all immunosuppressive therapy, except anti-CD40mAb, was discontinued 2 months after pig kidney transplantation. During follow-up for a further 2 months, no clinical or histopathological features of rejection were observed [133]. Nevertheless, we have seen antibody-mediated rejection on occasions when the rapamycin level fell to subtherapeutic levels.

One important observation made recently is that, when proteinuria is present (which may be an early sign of antibody-mediated rejection), therapeutic mAbs may be lost in the urine, thus exposing the xenograft to rejection [77]. Furthermore, there is some evidence that an infectious microorganism in a xenograft (e.g., pyelonephritis) may induce an immune response, resulting in rapid rejection as has been well-documented in ABO-incompatible kidney allotransplantation [134, 135].

## Comment

Throughout the early years covered by this brief report, researchers searched for other agents that might suppress the production of anti-pig antibodies. These agents included 1) various known immunosuppressive agents [30], 2) drugs used in other conditions but thought to have immunosuppressive properties [136–142], 3) new monoclonal antibodies directed towards depletion of plasma cells [143], 4) anti-idiotypic antibodies [144, 145], and 5) agents that influence expansion of T regulatory cells, but the majority proved unworthy or unnecessary of inclusion in the immunosuppressive regimen. An important observation was that prolonged treatment with bortezomib (a proteosome inhibitor) to patients who were highly sensitized to HLA had only a minimal effect in reducing anti-pig antibody levels [142].

Two agents that might well be valuable when xenotransplantation is introduced into the clinic are 1) atorvastatin [140, 141], whose anti-inflammatory effect could be of value. (As only tablets were available to us, we found it difficult to administer it successfully to NHPs.) and 2) alemtuzumab - but administering it to NHPs has several major limitations [146].

With the aim of protecting their cells from the adaptive immune response, gene-editing of the organ-source pigs has been explored. This included producing pigs that secreted CTLA4-Ig [147, 148] and pigs in which Major Histocompatibility Complex (MHC) Class II expression had been downregulated [149].

The level of CTLA4-Ig in the blood in pigs expressing CTLA4-Ig was approximately 10-fold higher than the therapeutic level in humans being treated with the agent [147]. Although this demonstrated the success of the gene-editing, the pigs were rendered immunocompromised and developed infections at a relatively young age for which they required euthanasia. This clearly precluded them from acting as sources of organs and from breeding. Furthermore, we concluded that maintaining the correct level of immunosuppression after organ transplantation in NHPs would also be difficult. However, others have successfully expressed CTLA4-Ig in the pancreatic islets [150, 151] although this limited expression may not be sufficient to protect against T cell-mediated rejection. MHC-Class II-knockdown was successful in reducing the T cell response and is worthy of further exploration [65, 149].

The currently available gene-edited pigs that are TKO and also express multiple human proteins [3, 76] would appear to be sufficient for clinical trials to be undertaken today. Future gene-editing may include the introduction of HLA-E and G [151], PD-L1 [152, 153], and MHC Class 1 knockout [154] or MHC Class II modification [155, 156].

Although gene-editing of pigs has been the major factor that has enabled progress to be made in pig organ xenotransplantation in NHPs, the introduction of immunosuppressive agents directed towards blockade of the CD40/CD154 co-stimulation pathway must not be underestimated. Successful clinical pig organ transplantation will require a combination of judicious gene-editing and the administration of an effective, but not excessive, immunosuppressive regimen. We suggest that selecting recipients with low levels of anti-pig IgM and preferably no IgG will contribute to success.

## Abbreviations

ATG, anti-thymocyte globulin; Gal, galactose-α1,3-galactose; GTKO, α1,3-galactosyltransferase gene-knockout; mAb, monoclonal antibody; MMF, mycophenolate mofetil; NHP, nonhuman primate; TKO, triple-knockout (i.e., with deletion of expression of the three known pig carbohydrate xenoantigens against which humans have natural antibodies.).
